# A Manual of Minor Surgery and Bandaging

**Published:** 1886-12

**Authors:** 


A MANUAL OF MINOR SURGERY AND BANDAGING. *For the Use of House-surgeons, Dressers and Junior Practitioners. By* CHRISTOPHER HEATH, F.R.C.S. *Eighth Edition; pp. 360, 12mo. Philadelphia:* P. BLAKISTON, SON & COMPANY. 1886.

The date of the preface to this edition is July, 1886. In this preface the author makes only two statements, viz., that the book is intended only for beginners, and that he "can only feel flattered," that the book is favorably mentioned in a certain popular novel. The first edition appeared in 1861, and the reader will to-day be as unable as the author apparently is to give a reason for the appearance of this edition. The chapter on antiseptics is enough to condemn the book, which, in the light of modern surgery, cannot fail to do harm whenever it falls into the hands for which it is stated that it is intended, viz., "beginners."

